# Effect of premedication on lesion detection rate and visualization of the mucosa during upper gastrointestinal endoscopy: a multicenter large sample randomized controlled double-blind study

**DOI:** 10.1007/s00464-018-6077-4

**Published:** 2018-03-23

**Authors:** X. Liu, C. T. Guan, L. Y. Xue, S. He, Y. M. Zhang, D. L. Zhao, Y. Li, F. G. Liu, J. Li, Y. F. Liu, A. S. Ling, W. Q. Wei, G. Q. Wang

**Affiliations:** 1 Department of Endoscopy, National Cancer Center/Cancer Hospital, Chinese Academy of Medical Sciences and Peking Union Medical College, 17 Panjiayuan, Chaoyang District, Beijing, 100021 China; 2 Department of Epidemiology, National Cancer Center/Cancer Hospital, Chinese Academy of Medical Sciences and Peking Union Medical College, 17 Panjiayuan, Chaoyang District, Beijing, 100021 China; 3 Department of Pathology, National Cancer Center/Cancer Hospital, Chinese Academy of Medical Sciences and Peking Union Medical College, Beijing, 100021 China; 4 Department of Cancer Prevention and Treatment Center, People’s Hospital, Feicheng, 271600 China; 5 Department of Gastroenterology, People’s Hospital, Linqu, 262600 China; 6 Department of General Internal Medicine, People’s Hospital, Dongping, 271500 China; 7 Department of Cancer Early Detection and Treatment, Cancer Hospital, Yanting, 621600 China; 8 Department of Endoscopy, People’s Hospital, Hebi, 458030 China; 9 Department of Gastroenterology, The First People’s Hospital, Anqing, 246003 China

**Keywords:** Lesion detection rate, Premedication, Pronase, Simethicone, Visualization

## Abstract

**Background:**

Premedication in upper gastrointestinal endoscopy for higher lesions detection rate has not been well studied so far. This study aimed to confirm whether premedication could improve the detection rate of early cancer or precancerous lesions and mucosal visibility.

**Method:**

From July 2015 to December 2015, 7200 participants from 6 centers were screened by endoscopy with one of the 4 following premedications randomly: (1) water (group D); (2) pronase (group A); (3) simethicone (group B); (4) pronase and simethicone (group C). Early cancer and precancerous lesions detection rates were taken as the primary endpoints, and mucosal visibility was taken as the secondary endpoint. They were compared among four groups to determine different premedication effects in terms of different anatomical sites. Trial was registered at Chinese Clinical Trial Registry; the registration number is ChiCTR-IOR-17010985.

**Results:**

The upper gastrointestinal overall precancerous lesion detection rates among four groups were 8.7, 8.4, 10.0, and 10.3%, the overall early cancer detection rates were 1.3, 1.4%, 1.5, and 1.6%, both without significant difference (*p* = 0.138 and 0.878). However, the visibility score distributions between control group (D) and premedication groups (A, B, and C) were all statistically significant, with all anatomical sites *p* values < 0.001. Subgroup analyses, from 2 centers without screening before, also showed significant difference in esophageal (3.9, 3.3, 4.5, and 8.4% with *p* = 0.004) and overall (7.0, 5.5, 7.3, and 12.0% with *p* = 0.004) precancerous lesion detection rate.

**Conclusions:**

Premedication with pronase and simethicone may not increase lesion detection rates but could significantly increase the upper gastrointestinal mucosal visibility.

## Introduction

Favorable esophageal and gastric mucosal visibility during upper gastrointestinal (UGI) endoscopy is essential for high-quality examination and minute malignant lesions identification. Mucus and foam can reduce the mucosal visibility and may increase the frequency of missing clinically relevant lesions. Therefore, premedication that reduces mucus and foam may be an effective strategy to improve the visibility of minimal lesions, such as early cancer or precancerous lesions, and could increase detection rates.

In many endoscopy centers, simethicone has been reported as an effective defoaming agent [1–3]. Pronase, a mixture of proteolytic enzymes, was first isolated in 1962 from the culture filtrate of *Streptomyces griseus*, which was already used as a mucolytic agent to improve visualization of the mucosa by its mucolytic effect [4–8]. However, the effect of premedication on the detection rate of early cancer or precancerous lesions has not been well investigated before. The aim of this study was to explore whether premedication could improve the detection rate of early cancer or precancerous lesions and mucosal visibility.

## Materials and methods

### Participants

From July 2015 to December 2015, a consecutive series of participants from the following six high UGI cancer incidence centers: Feicheng (Center I), Dongping (Center II), Linqu (Center III), Hebi (Center IV), Anqing (Center V), and Yanting (Center VI), who attended the Rural Cancer Early Detection & Treatment Program (RCEDTP) in China for UGI endoscopy screening, were enrolled in the randomized controlled trial. Inclusion and exclusion criteria were consistent with criteria from RCEDPT. Inclusion criteria were as follows: (1) aged between 40 and 69 years; (2) volunteered in the trial, and signed the informed consent. Exclusion criteria were as follows: (1) complete pyloric obstruction; (2) active gastrointestinal bleeding; (3) history of upper gastrointestinal surgery; (4) severe heart, liver, and kidney disease and unfit for endoscopy; (5) pregnant and lactating women; (6) has participated in other clinical studies within a month; (7) psychosis and severe neurosis; (8) allergic to drugs taken during the test.

Informed consent was signed by all participants prior to enrollment. This clinical trial has been permitted by the ethics committee in Cancer Hospital, Chinese Academy of Medical Sciences, Peking Union Medical College (Approval Number: 15-059/986; Scheme No. CH-END-001). The clinical trial is registered at Chinese Clinical Trial Registry, and the registration number is ChiCTR-IOR-17010985.

### Sample size, randomization, and study design

We calculated different sample sizes according to the lesion detection rates of the esophagus, cardia, and stomach, respectively. Final sample size for the study was determined based on the gastric lesion detection rate, which is reported to be the lowest among the three anatomical sites. 0.85 and 0.05 were taken as the power of the test and α in our study, respectively. Since multiple comparisons among four groups were needed in our study, we adjusted α (0.05/6) when calculating the sample size. Because of low detection rate of gastric lesion in the population, we considered at least 1.5% increase of it as clinically relevant difference, between Group C and Group D. Based on these parameters, we got the total sample size of 7200 cases.

**Fig. 1:**
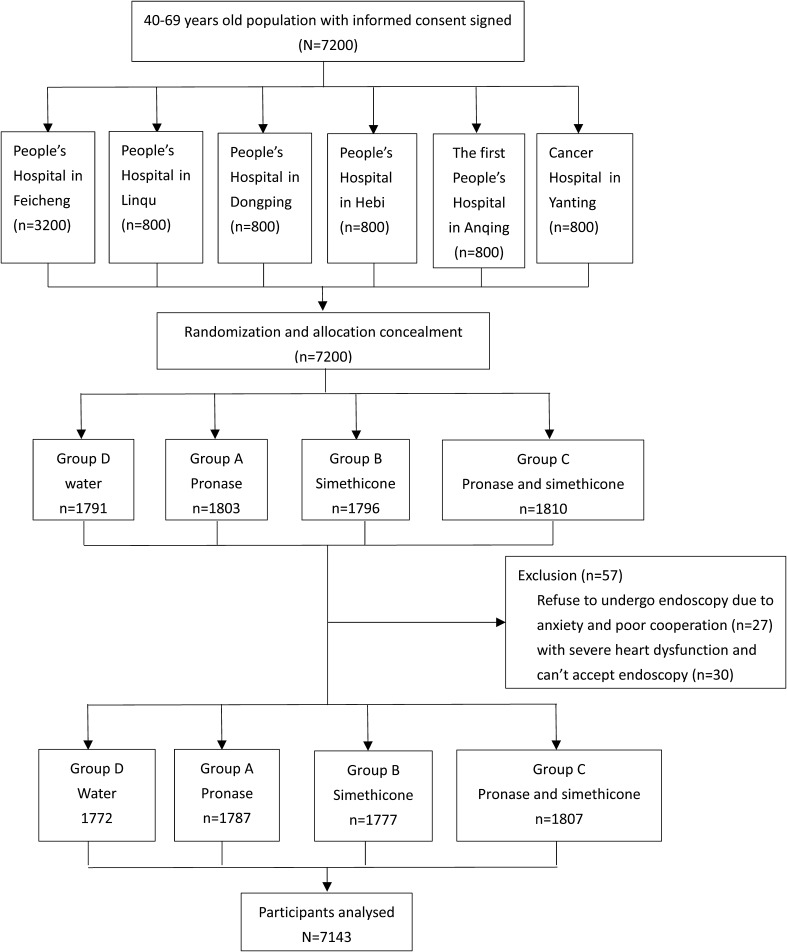
Flow diagram of sample distribution

Based on the available participants of each center, 3200 samples were allocated to Center I, and 800 for each of the other centers (Fig. 1). Participants in each center were randomly assigned to four study groups: (1) 100 mL of warm water containing 20,000 units of pronase and 1 g of NaHCO_3_ at 40 °C (group A); (2) 100 mL of warm water containing 80 mg of simethicone at 40 °C (group B); (3) 100 mL of warm water containing 20,000 units of pronase, 80 mg of simethicone, and 1 g of NaHCO_3_ at 40 °C (group C); and (4) 100 mL of warm water (group D) at 40 °C. A total of 7200 random numbers were generated by SAS 9.2 statistical software for each participant. The participant ID number, the corresponding random number, and the corresponding group information were written on the same card, with the latter two in a sealed cover. After cards were manufactured, they were delivered to the study centers. All participants signed the informed consent of clinical trial before getting a card. The hidden information on the card was revealed by a designated person who did not enter the examination room and was not involved in the endoscopic procedures. Then, participants were administered drugs of the corresponding group. Both the study participants and the endoscopists remained blinded to the premedication drugs during the study.

### Premedication procedures, endoscopic examination, and data evaluation

**Fig. 2:**
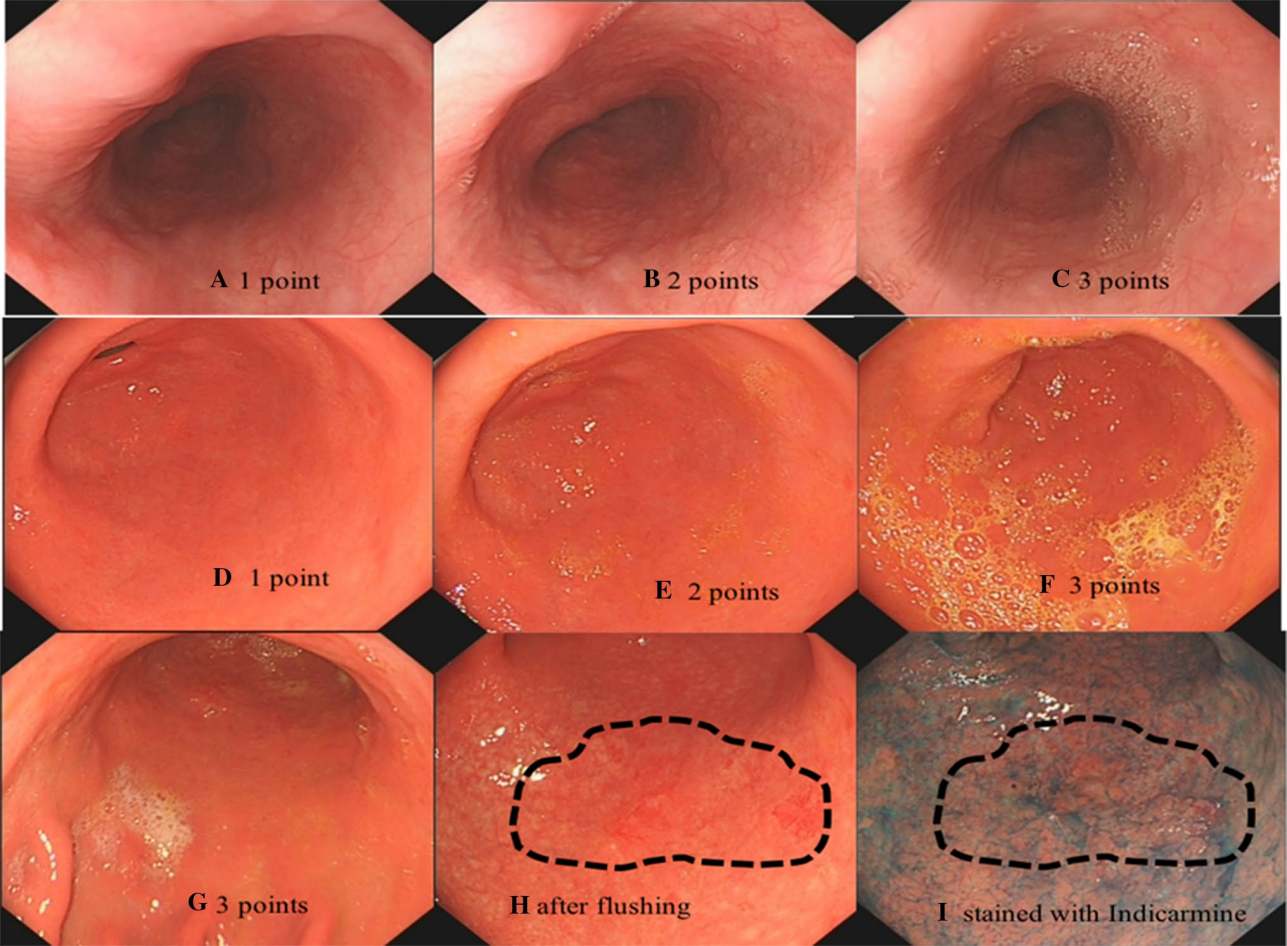
Visibility score images. **A**–**C** Visibility score of esophagus from one to three points, one point means no adherent mucus, two points mean mild mucus not obscuring vision, three points mean a large amount of mucus obscuring vision and requiring water to clear. **D, E** Visibility score of antrum from one to three points. **G** Three points of gastric body. **H** A hidden early gastric cancer lesion was found after flushing. **I** The early gastric cancer lesion stained with Indigo carmine

The premedication was administered orally approximately 20 min before endoscopy. After premedication, the participants were given intravenous fluids, and guided to the examination room where the UGI endoscopy was performed. Participants underwent routine UGI endoscopic examinations using GIF-H260 video endoscope (Olympus, Tokyo, Japan). All procedures were video recorded. After the removal of excess gastric solution, the visibility scores were evaluated separately at esophagus, cardia, fundus, gastric body, and antrum by the endoscopists. The visibility scores ranged from 1 to 3 [7] (Fig. 2): score 1 is no adherent mucus; score 2 is mild mucus but not obscuring vision; score 3 is a large amount of mucus, obscuring vision, and requiring water to clear. NBI and chromoendoscopy with Lugol’s iodine was routinely done in the esophagus in all participants, whereas Indigo carmine stain was used in the stomach if there were suspicious lesions. Endoscopic biopsies were required when lesions were suspected. The visibility scores and the location of biopsy were recorded, and the pathological diagnoses were checked afterwards.

Drug allergy was the major adverse events that happened in this study. Complications include bleeding, perforation, and aspiration pneumonia. Lesions include early cancer and precancerous lesions from esophageal, cardia, and stomach. Cardia area refers to the upper and lower range of 5 cm, respectively, of esophagogastric junction (EGJ) according to Siewert’s criteria. “Early cancer” means lesions with cancer cells detected pathologically, and endoscopic features meet the criterion of superficial lesions of digestive tract cancer according to Paris classification. “Precancerous lesions” were defined as lesions with low-grade intraepithelial neoplasia or high-grade intraepithelial neoplasia.

### Quality control

UGI endoscopy was performed by four experienced endoscopists in Center I and two in each of the other centers. All endoscopic procedures were done under sedation, with intravenous anesthesia given by professional anesthetists.

To minimize interobserver variation, frequent study meetings were held. Endoscopists and supervisors were provided with standardized instructions for standard operation and evaluation. All endoscopists and supervisors were required to have experience of performing more than 3000 and 5000 UGI endoscopic procedures, respectively. The supervisors reviewed 30% of the test video records randomly, and the data were considered reliable only when the error proportion fell within 5%.

The maximal activity environment of pronase is pH 6–8 [7], temperature 20–40 °C, and reaction time 10–30 min [9]. In order to achieve the best effect, a neutralizer (NaHCO_3_) was added to neutralize the acidity of the gastric juice in Group A and Group C, the temperature of the premedication was maintained at 40 °C, and participants were instructed to took premedication 20 min before UGI endoscopy. Meanwhile, pure water was used to flush if clearing mucus and foam was needed during the examination. This could avoid the interference of the intraoperative flushing on the judgement of the visibility score.

### Statistical analysis

Per-protocol analysis was done with 7143 participants included in the analyzed dataset (Fig. 1). The primary endpoints were UGI lesion detection rates (including early cancer and precancerous lesions), exploring the efficiency of preoperative medication. Visibility scores were the secondary endpoints. SAS 9.2 was used for data analysis. First, participants’ baseline characteristics were described and their differences were tested by analysis of variance and *χ*^2^ test. After confirming no significant difference in baseline information among different groups, *χ*^2^ tests were used to compare primary endpoints, and Kruskal–Wallis rank-sum tests were used for secondary endpoints comparison. For the potential reason of low baseline detection rates resulted from some centers’ secondary endoscopy surveillance leading to the negative results, the data from Center III and Center IV were merged for further subgroup analysis, and the aforementioned statistical analysis procedures were repeated.

## Results

### Baseline participant characteristics

The baseline characteristics are shown in Table 1. No significant differences have been observed among the four groups in terms of age, weight, height, sex, smoking, drinking, family history of cancer, and chronic diseases such as hypertension, diabetes, heart disease, and respiratory disease. Endoscopy time and volume of flushing water were the only significant differences among the four groups. These two variables could be considered as intermediate variables and associated with premedication, so the baseline was still balanced among four groups. Neither serious complications related to the endoscopic operation nor adverse events that need hospitalization were reported in our study.

**Table 1:** Baseline information description

	Mean ± SD/*N* (%)				*P* value
	Group D (Water)	Group A (Pronase)	Group B (Simethicone)	Group C (Pronase and simethicone)	
Age^a^	53.6 ± 7.7	53.9 ± 7.6	53.5 ± 7.6	53.7 ± 7.7	0.273
Weight (kg)^a^	63.0 ± 10.1	63.5 ± 9.9	63.5 ± 10.0	63.3 ± 10.2	0.485
Height (cm)^a^	162.1 ± 7.0	162.1 ± 7.1	162.2 ± 7.0	162.3 ± 7.1	0.787
Endoscopy time (min)^a^	10.3 ± 4.8	9.8 ± 4.8	9.0 ± 4.9	8.9 ± 4.9	< 0.001
Flushing water volume (ml)	170.9 ± 101.2	131.0 ± 90.1	63.4 ± 62.7	43.2 ± 54.2	< 0.001
Gender (male)	733 (41.4)	746 (41.7)	748 (42.1)	757 (41.9)	0.976
Smoking (Y)	343 (19.2)	342 (19.2)	334 (18.8)	339 (18.8)	0.944
Drinking (Y)	306 (17.3)	339 (19.0)	310 (17.4)	321 (17.7)	0.507
Hypertension (Y)	83 (4.7)	87 (4.9)	78 (4.4)	81 (4.5)	0.906
Diabetes (Y)	34 (1.9)	24 (1.3)	30 (1.7)	24 (1.3)	0.417
Heart disease (Y)	10 (0.6)	15 (0.8)	9 (0.5)	7 (0.4)	0.327
Respiratory diseases (Y)	1 (0.1)	8 (0.4)	6 (0.3)	3 (0.2)	0.076
Family history of cancer (Y)	353 (19.9)	344 (19.3)	327 (18.4)	322 (17.8)	0.391
Other related diseases (Y)	6 (0.3)	3 (0.2)	4 (0.2)	3 (0.2)	0.701

^a^Age, weight, height, and endoscopy time are quantitative variables, we use mean and standard deviation to describe the central and dispersion tendency. The other variables showed the positive numbers and percentages

### Primary endpoints

The detection rate of early cancer and precancerous lesions of esophagus, cardia, and stomach had no significant difference among the four groups, respectively (Table 2). Combining the lesions of esophagus, cardia, and stomach, results showed that the overall (participants who had lesions in different sites simultaneously were counted only once) detection rates of early cancer (1.3, 1.4%, 1.5 and 1.6%, *p* = 0.878) and precancerous lesions (8.7, 8.4, 10.0, and 10.3%, *p* = 0.138) among the four groups also were not significantly different.

**Table 2:** UGI lesion detection rate comparison

	Detection rate				*P* value
	Group D (Water) (%)	Group A (Pronase) (%)	Group B (Simethicone) (%)	Group C (Pronase and simethicone) (%)	
Esophagus					
Precancerous lesions	5.6	5.5	6.2	7.1	0.166
Early cancer	0.7	0.8	0.9	1.0	0.829
Cardia					
Precancerous lesions	1.8	1.6	2.1	1.6	0.681
Early cancer	0.5	0.3	0.2	0.3	0.715
Gastric					
Precancerous lesions	1.6	1.8	2.3	2.0	0.511
Early cancer	0.2	0.3	0.4	0.3	0.649
Overall					
Precancerous lesions	8.7	8.4	10.0	10.3	0.138
Early cancer	1.3	1.4	1.5	1.6	0.878

### Secondary endpoints

The visibility score distributions of esophagus, cardia, fundus, gastric body, and antrum were all significantly different among the four groups (Table 3). To confirm the efficacy of each premedication, visibility score distribution comparisons were done between every two groups. Premedication groups (A, B, and C) had better visibility scores than the control group (D) at each anatomical site of the UGI tract. Both group C and group B had significant difference compared with group A at each anatomical site of the UGI tract. Statistically significant differences between groups C and B were only found in fundus, gastric body, and antrum.

**Table 3:** Visibility score distributions comparison of esophagus, cardia, fundus, gastric body, and antrum

	Number (%)				*P* value					
	Group D (Water)	Group A (Pronase)	Group B (Simethicone)	Group C (Pronase and simethicone)	D vs. A	D vs. B	D vs. C	A vs. B	A vs. C	B vs. C
Esophagus					< 0.001	< 0.001	< 0.001	< 0.001	< 0.001	0.228
1	120 (6.8)	268 (15.0)	792 (44.6)	917 (50.7)						
2	913 (51.5)	933 (52.2)	798 (44.9)	675 (37.4)						
3	739 (41.7)	586 (32.8)	187 (10.5)	215 (11.9)						
Cardia					< 0.001	< 0.001	< 0.001	< 0.001	< 0.001	0.057
1	88 (5.0)	214 (12.0)	920 (51.8)	1055 (58.4)						
2	788 (44.5)	909 (50.9)	740 (41.6)	626 (34.6)						
3	896 (50.5)	664 (37.1)	117 (6.6)	126 (7.0)						
Fundus					< 0.001	< 0.001	< 0.001	< 0.001	< 0.001	< 0.001
1	103 (5.8)	218 (12.2)	896 (50.4)	1124 (62.2)						
2	582 (32.8)	750 (42.0)	699 (39.3)	534 (29.6)						
3	1087 (61.4)	819 (45.8)	182 (10.3)	149 (8.2)						
Gastric body					< 0.001	< 0.001	< 0.001	< 0.001	< 0.001	< 0.001
1	121 (6.8)	315 (17.6)	821 (46.2)	1157 (64.0)						
2	705 (39.8)	829 (46.4)	786 (44.2)	541 (29.9)						
3	946 (53.4)	643 (36.0)	170 (9.6)	109 (6.1)						
Antrum					< 0.001	< 0.001	< 0.001	< 0.001	< 0.001	< 0.001
1	375 (21.2)	656 (36.7)	1271 (71.6)	1492 (82.6)						
2	721 (40.7)	645 (36.1)	422 (23.7)	240 (13.3)						
3	676 (38.1)	486 (27.2)	84 (4.7)	75 (4.1)						

### Subgroup analyses

The data of Center III and Center IV, which participated RCEDTP for the first time, were analyzed together (Table 4). Results showed significant difference in esophageal (3.9, 3.3, 4.5, and 8.4% with *p* = 0.004) and overall (7.0, 5.5, 7.3, and 12.0% with *p* = 0.004) precancerous lesions detection rate.

**Table 4:** UGI lesion detection rate in Center IV and Center III

	Detection rate				*P* value
	Group D (Water) (%)	Group A (Pronase) (%)	Group B (Simethicone) (%)	Group C (Pronase and simethicone) (%)	
Esophagus					
Precancerous lesions	3.9	3.3	4.5	8.4	0.004^a^
Early cancer	0.5	0.5	0.8	1.5	0.488
Cardia					
Precancerous lesions	0.5	0	0.5	0.2	0.497
Early cancer	0.5	0	0	0.2	0.195
Gastric					
Precancerous lesions	2.9	2.3	2.3	3.7	0.566
Early cancer	0.5	0.5	0.8	1.0	0.894
Overall					
Precancerous lesions	7.0	5.5	7.3	12.0	0.004^b^
Early cancer	1.6	1.0	1.5	2.7	0.295

^a^The paired comparison p values (C VS. D, A, and B) were 0.010, 0.002, and 0.0028, respectively, adjusted *α* = 0.05/6

^b^The paired comparison p values (C VS. D, A, and B) were 0.017, 0.001, and 0.0024 respectively, adjusted *α* = 0.05/6

## Discussion

Pronase is a mucolytic agent used for digesting esophageal mucus, and simethicone is a silicone-based non-absorbable material that causes bursting of gas bubbles by reducing surface tension. Therefore, both the drugs can improve the mucosal visibility of UGI endoscopy [6, 10, 11]. Previous studies on UGI endoscopy premedication have focused on mucosal visibility [4, 7, 9, 12–15], and a recently published double-blind randomized trial has proved that both pronase and simethicone can result in better visibility [16]. However, the detection rate improvement on UGI early cancer and precancerous lesions through oral premedication has not been well evaluated.

Our study validated the mucosal visibility effect of UGI endoscopy premedication again with a larger sample in multi-centers. The mucosal visibility of the pronase, simethicone, and combination group was better than that of the control group (Table 3). In addition, it was also found that the mucosal visibility of the simethicone group was better than that of the pronase group. This may be because of more conspicuous effect on visibility improvement from simethicone. Compared to the control group, simethicone made the percentage of “Score 1” increase by about 38–50%. However, pronase only increased about 6–15%. The additive effect of these two agents could be confirmed in the combination group. Nevertheless, no significant difference was found in esophagus and cardia between the combination and simethicone groups. Since foams were the major reason of visibility interference in esophagus and cardia, the effect of pronase was not so obvious there. But as anti-foaming agent, simethicone was probably able to significantly improve mucosal visibility. That means in esophagus and cardia, simethicone plays the major role to improve visibility, and this is consistent with our study finding. While in fundus, gastric body, and antrum, other than foam, mucus is also an important factor influencing visibility. Therefore, mucolytic agent could also play a significant role [6]. We assume this is the reason for the difference in visibility score distributions in fundus, gastric body, and antrum between the combination and simethicone groups in our study. For the whole upper gastrointestinal tract, it was hard to get the best mucosal visibility only with anti-foaming agents. Hence, to achieve the best visibility of the mucosa, the mucolytic agent needed to be combined with anti-foaming agents.

It was expected that the lesion detection rate would increase after improvement in the UGI endoscopic visibility through premedication. However, for esophagus, cardia, stomach, and overall lesion detection rates, there was no significant difference among the four groups.

This multicenter trial was based on the RCEDTP in China. The periodic interval of this program is 5 years, since the consensus is that 5-year periodic interval is sufficient for the occurrence and development of a disease. A previously screened area would undergo endoscopy surveillance every 5 years. Hence, the following endoscopy detections would cover many participants who previously had the primary screening. After the primary screening discovered early cancer or precancerous lesions of UGI, they were recommended to be treated timely. Although 5-year periodic interval was sufficiently long for the new lesions to occur, the baseline lesion detection rate in the secondary surveillance rural centers was significantly low compared to the primary screening. As the sample size for the study was determined based on the primary screening detection rate, inclusion of the participants who underwent secondary surveillance study could have resulted in lower baseline lesion detection rates seen in our study. Among all the centers, rural areas of Center I included in the study were secondary surveillance areas, so the baseline lesion detection rate of People’s Hospital in Center I was lower compared with the previous data. Meanwhile, the sample number People’s Hospital in Center I was 3200, accounting for nearly half of the entire sample. As a result, the relatively low lesion detection rate strongly affected the overall baseline detection rate of the trial, and finally leading to non-significant difference. Actually, except Center III and Center IV, which involved RCEDTP for the first time, the other centers more or less contained secondary surveillance participants.

When designing the trail, 1.5% increase of gastric lesions detection rate was selected as the meaningful clinical difference. The determination of this parameter was based on the primary screening detection rate. However, in this study neither the detection rates of early cancer nor the precancerous lesions were significantly improved. In addition to the inclusion of secondary surveillance participants in those centers, which lead to lower incidence of the UGI lesions, the overestimate clinical meaningful difference cut-off of 1.5% may be another potential reason. In other words, baseline detection rate determines improvement potential. Translating this into clinical practice, for experienced endoscopists, lesions could be found through proper water flushing despite no premedication. In fact, their baseline detection rate may be very close to peak, and the premedication effect may have limited effect for them, so this could be another potential reason for the negative results in our study.

To exclude the impact of secondary endoscopy surveillance, the data of each center were analyzed separately. By analyzing the data of Center III and Center IV together, it was found that the esophagus and overall detection rate had statistical difference in precancerous lesions. Since cardia and stomach did not have detection rate difference among four groups, we think that the overall detection rate difference mainly resulted from esophagus. Even though it was just a subgroup analysis, we want to show its significance here. Firstly, comparing esophagus precancerous lesion detection rate in group D (Tables 2, 4), the subgroup analysis got lower detection rate than the total (3.9 vs. 5.6%). This may be because of real lower esophageal lesions incidence or relatively poorer technique in finding esophageal lesions. Then comparing esophagus precancerous lesion detection rate in group C, the detection rates were opposite (8.4 vs. 7.1%). This phenomenon excluded the possibility of lower incidence in two subgroup centers, and actually Center III and Center IV should have higher incidence because of the primary screening. So the only reason for the phenomena is relatively poorer technique in these two centers, which is consistent with our above imagination. That is, for experienced endoscopists, premedication of pronase combined with simethicone may have limited significance, but for inexperienced ones it may be a good choice. However, anyway as it was just a subgroup analysis, in this research we could not draw the final conclusion.

The European Society of Gastrointestinal Endoscopy (ESGE) guidelines have recommended procedure time for standard endoscopy, [17] and our research also studied the premedication effect on the endoscopic inspection time. The groups with better visibility scores had lower endoscopic inspection time, probably because of less need for flushing. In general clinical practice in China, most endoscopic examinations are not completely under sedation, hence less flushing and shorter inspection time can reduce the discomfort for patients. However, the difference of examination time was only about 1 min, which seems without enough clinical relevance.

Our study has some limitations. First, because of the unbalanced sample distribution among six centers and unexpected low baseline detection rates, the negative result of Center I highly affected and even determined the total result. Second, endoscopies were performed by selected highly skilled endoscopists, the results may not be generalizable to community endoscopists. For experienced endoscopists, the UGI lesions detection rates did not increase obviously with premedication of pronase and simethicone. It needs further research whether this conclusion could be expanded to all endoscopists. Third, despite having specialized staff supervising the study, it was hard to ensure that the premedication was given at 20 min before the study, which might have affected their activity and effect. Fourth, according to the CONSORT guidelines, a written card with a covered part is not considered a correct allocation concealment, so our allocation concealment may have had some limitations.

In conclusion, this was the first large sample multicenter randomized controlled double-blind study to show the influence of different premedication methods before UGI endoscopy on the UGI lesion detection rate and visibility. The combination agents group had a better mucosal visibility compared with single agent or control group. Besides, premedication with simethicone can better improve the visibility than pronase. However, improved mucosal visibility may not obviously increase lesion detection rates for experienced endoscopists. Whether it makes sense or not for inexperienced endoscopists in higher detection rate areas needs further study.
